# A Systematic Review and Meta-Analysis of Preoperative Characteristics and Postoperative Outcomes in Patients Undergoing Endoscopic Spine Surgery: Part I Endoscopic Microdiscectomy

**DOI:** 10.3390/jcm14196757

**Published:** 2025-09-24

**Authors:** Long Di, Andrew Wang, Kate E. Stillman, Lauren K. Tierney, Solomon G. Jackson, Andrew J. Sasser, Alexander Valecillo, Tyler Cardinal, Seth Tigchelaar, Adham M. Khalafallah, Gregory Basil

**Affiliations:** Department of Neurological Surgery, University of Miami Miller School of Medicine, 1120 NW 14th Street, Miami, FL 33136, USA; andrew.wang@jhsmiami.org (A.W.); kes273@med.miami.edu (K.E.S.); lkt32@miami.edu (L.K.T.); sgj39@med.miami.edu (S.G.J.); ajs5730@med.miami.edu (A.J.S.); alexandervalecillo@icloud.com (A.V.); sst98@miami.edu (S.T.); amk362@miami.edu (A.M.K.); gbasil@med.miami.edu (G.B.)

**Keywords:** endoscopic lumbar discectomy, full endoscopic lumbar discectomy (FELD), biportal endoscopic lumbar discectomy (BELD), minimally invasive spine surgery (MIS), endoscopic spine surgery, meta-analysis, systematic review

## Abstract

**Background/Objectives**: Rates of degenerative spinal pathology are increasing, driving interest in minimally invasive surgical (MIS) techniques that facilitate faster recovery. Full endoscopic lumbar discectomy (FELD) and biportal endoscopic lumbar discectomy (BELD) offer reduced tissue disruption, but comparative outcomes versus non-endoscopic MIS and optimal patient selection remain unclear. This systematic review examines pre-operative characteristics and post-operative outcomes of endoscopic lumbar microdiscectomy (ELMD) compared to MIS and open techniques. **Methods**: A PRISMA-guided search of PubMed, Embase, Scopus, and hand searches through 31 September 2024 identified studies on lumbar spinal surgery using endoscopic techniques, restricted to level 1a/b and 2a evidence. Articles were subgrouped by surgery type, with this analysis focusing on ELMD. Data extraction included risk-of-bias assessment, and meta-analysis was performed using multivariate mixed-effects regression. Pre-operative patient characteristics and post-operative outcomes for endoscopic lumbar microdiscectomy (ELMD) were directly compared to both open microdiscectomy and minimally invasive non-endoscopic microdiscectomy (MIS) techniques. Within the ELMD cohort, we further analyzed differences between full endoscopic (FELD) and biportal endoscopic (BELD) approaches, as well as between transforaminal and interlaminar access routes. **Results**: Of 6891 articles, 5469 unique titles/abstracts were screened, yielding 87 studies (3238 patients) for final synthesis. Compared to open microdiscectomy, ELMD patients were more often male, younger, of lower BMI, and had more comorbidities. They typically presented with shorter symptom duration and predominant radiculopathy. ELMD was performed most at L3–L4 and L4–L5. Post-operatively, ELMD patients had significantly lower VAS Leg Pain scores at 1 day and 1 year and reduced recurrence rates. ELMD was associated with lower recurrence rates and correspondingly lower revision surgery rates, with dural tears and wound infections trending lower compared to open surgery. Compared to non-endoscopic MIS, pre- and post-operative characteristics were similar. BELD patients more often had longer symptom duration, motor weakness, and hyporeflexia than FELD patients. **Conclusions**: ELMD patients demonstrate favorable pain relief and reduced recurrence versus open surgery, with outcomes comparable to MIS. These findings support ELMD as a less invasive alternative within the MIS spectrum.

## 1. Introduction

Lumbar disk herniation (LDH) is a common degenerative spinal disorder with a prevalence of 1–3% of the population. LDH is most common in working individuals 30 to 50 years of age, with the prevailing belief that progressive mechanical insults such as heavy physical loading, bending, lifting, or twisting contribute to disk [1]. However, several additional key risk factors have been identified, including smoking, obesity, and old age [2]. As life expectancy increases and obesity rates continue to rise [3,4], the demand for effective surgical interventions has grown, particularly for minimally invasive techniques that prioritize faster recovery, reduced postoperative morbidity, and quicker return to work.

The microscopic lumbar microdiscectomy has remained the standard of care for patients who fail conservative management. This minimally invasive surgery (MIS) has arisen as an effective adjunct to decrease postoperative pain, muscle trauma, and hospital length of stay while achieving comparable outcomes [5]. Within MIS, endoscopic lumbar microdiscectomy (ELMD)—which includes both full endoscopic lumbar microdiscectomy (FELD) and endoscopic-assisted, or biportal endoscopic lumbar discectomy (BELD)—offers significant advantages such as reduced blood loss, shorter hospitalization, and faster return to daily activities [6,7,8]. However, despite these benefits, concerns remain regarding the learning curve, technical limitations, and potential complications such as dural tears and residual disk fragments [9,10]. Considering these facts, understanding patient selection, surgical technicalities, and post-operative outcomes for an endoscopic approach is of critical importance.

Here, we present a scoping systematic review to synthesize existing evidence on the use of endoscopic lumbar microdiscectomy for the treatment of LDH. We examine and compare both pre-operative characteristics and post-operative outcome data of patients undergoing endoscopic lumbar microdiscectomy (both FELD and BELD) compared to traditional open surgery. We also compare FELD and BELD techniques as well as transforaminal and interlaminar approaches to the disk space.

## 2. Materials and Methods

### 2.1. Summary

We performed a systematic review according to the Cochrane Handbook for Systematic Review of Interventions and the PRISMA (Preferred Reporting Items for Systematic Reviews and Meta-Analyses) standard. 

### 2.2. Data Source and Literature Searches

We developed our search strategy in consultation with an expert librarian (Calder Library, University of Miami, Miami, FL, USA) using keywords associated with “endoscopy”, “minimally invasive”, “lumbar”, “microdiscectomy”, “decompression”, and “fusion”. We searched PubMed, Embase, and Scopus until 31 September 2024 and manually queried study references, but none included. To assess feasibility, a preliminary search was conducted in consultation with neurosurgical experts within the field.

### 2.3. Study Eligibility and Selection

Because the type of patients selected for lumbar surgery varies by country and the surgeon’s expertise, we sought to evaluate studies with details on post-operative outcomes compared to non-endoscopic MIS approaches and patient selection criteria for endoscopy. The reviewers (A.W. and K.E.S.) appraised the pertinent studies to determine eligibility. Studies were included if (1) minimally invasive endoscopy techniques were utilized in either microdiscectomy, decompression, or fusion surgeries; (2) surgery involved the lumbar spine; and (3) the study was of level 1 a/b and 2a evidence as defined by the Levels of Evidence from the Centre for Evidence-Based Medicine (CEBM), Oxford. No period or language restrictions were applied. Non-English language articles were to be translated through the publisher’s website or Google Translate (Google LLC, Mountain View, CA, USA). We excluded studies, out-of-scope review articles, commentaries, and conference abstracts without accompanying full articles. Articles were then subgrouped by type of surgery performed, with the current study focused on endoscopic microdiscectomy, and data abstraction and synthesis were then performed as detailed below.

### 2.4. Data Abstraction and Synthesis

**Figure 1:**
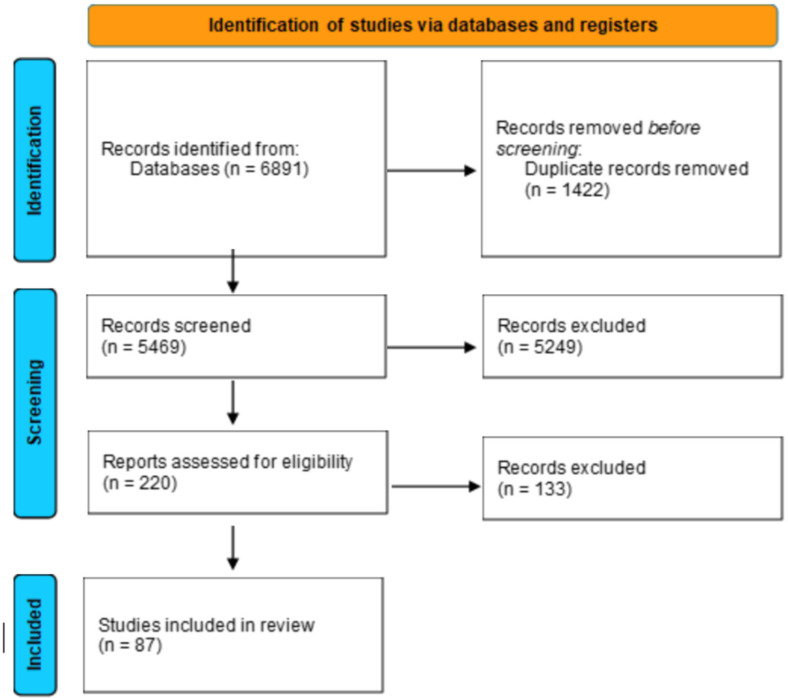
PRISMA flow diagram depicting study selection, inclusion, and exclusion criteria for this systematic review.

Our search strategy is shown in the PRISMA flowchart in Figure 1. Titles and abstracts were evaluated for initial screening. Full-text articles were assessed for eligibility on Rayyan version 2024 (Rayyan Systems Inc., Cambridge, MA, USA). Data were abstracted for all included studies. The reviewers (A.W. and K.E.S.) independently abstracted data from the included studies into a Microsoft Excel version 2016 spreadsheet (Microsoft, Redmond, WA, USA) and managed on EndNote (X9; Clarivate, Philadelphia, PA, USA). Information extracted included study year, study design, number of participants, demographics (e.g., gender, age, BMI, past medical or surgical history, smoking status, insurance), preoperative symptoms, length of preoperative symptoms, preoperative labs, levels of lumbar surgery, type of endoscopy technique, VAS leg and back pain scores at under a month and over month. Any disagreements in eligibility and abstraction were resolved through discussion (Cohen κ = 0.64). The primary outcomes of interest were early (postoperative day 1) and long-term (1 year) VAS leg pain scores and recurrence rates. Secondary outcomes included VAS back pain, rates of dural tear, wound infection, and revision surgery. These endpoints were selected to address our core research questions: whether ELMD provides superior short- and long-term pain relief, whether it reduces complication and recurrence rates, and how outcomes compare to open and MIS non-endoscopic approaches.

### 2.5. Assessment of Risk of Bias

The reviewers (A.W. and K.E.S.) independently assessed the methodological quality of each included study using the tool developed by ROBINS-I (Risk of Bias in Non-randomized Studies of Interventions) across 7 domains (confounding, selection bias, misclassification bias, performance bias, attrition bias, detection bias, and outcome reporting bias). Rated studies were given an overall summary assessment of low risk of bias if all the key domains were rated as low; moderate risk of bias if all the key domains were rated as low or moderate; serious risk of bias if at least 1 domain but not at critical risk of bias in any other domain; critical risk of bias if at least 1 domain; no information if there is no clear indication that the study is at serious or critical risk of bias and there is a lack of information in 1 or more key domains of bias. Any disagreement in abstraction or risk of bias assessments was resolved through discussion (Cohen κ = 0.88).

### 2.6. Analytical Plan

A mixed-methods approach was taken to gather study data. The review focused on differences in post-operative outcomes compared to non-endoscopic MIS approaches and cataloging ideal patient selection criteria for endoscopic lumbar surgery. Relationships between variables were described qualitatively. Abstracted information on study design, number of participants, and demographics was described as proportions or means. Study characteristics, patient demographics, and preoperative categorical variables were reported as counts or percentages with 95% confidence intervals. When possible, study-level preoperative data, along with postoperative pain scoring outcomes, were evaluated using multivariate mixed-effects regression modeling with odds ratio (OR) reporting by using Comprehensive Meta-Analysis Version 4 (Biostat Inc., Englewood, NJ, USA). Scoring agreements were expressed as a weighted Cohen κ coefficient (<0.20 as slight, 0.21–0.40 as fair, 0.41–0.60 as moderate, 0.61–0.80 as substantial, and >0.81 as almost perfect agreement). For all missing values, the data were censored for final analysis. 

## 3. Results

Of 6891 articles, 5469 unique articles were included for title abstract screening, of which 220 full-text microdiscectomy articles were evaluated. For the final synthesis, 87 microdiscectomy studies were included [11,12,13,14,15,16,17,18,19,20,21,22,23,24,25,26,27,28,29,30,31,32,33,34,35,36,37,38,39,40,41,42,43,44,45,46,47,48,49,50,51,52,53,54,55,56,57,58,59,60,61,62,63,64,65,66,67,68,69,70,71,72,73,74,75,76,77,78,79,80,81,82,83,84,85,86,87,88,89,90,91,92,93,94,95,96,97]. The majority of microdiscectomy studies were from Asia (75.8%), Europe (8.0%), and North America (6.9%), and were conducted between 2019 and 2024 (69.0%). Most studies were nonrandomized retrospective cohort studies (77.0%), which included a total of 3238 participants.

### 3.1. Pre-Operative Demographic and Clinical Data

The studies included a total of 3238 patients, of which a total of 2074 endoscopic lumbar microdiscectomies (both FELD and BELD), 1167 open lumbar microdiscectomies, and 249 MIS non-endoscopic microdiskectomies were included in the final statistical meta-analysis. The average age of the entire cohort was 45.8 ± 11.3, the average BMI was 24.5 ± 3.1, and the cohort consisted of more males (60.0%). The mean age of patients in the endoscopy group was significantly lower than that of the open group (48.2 vs. 52.7 years, *p* < 0.001, Table 1). Similarly, the mean BMI was significantly lower in the endoscopy group than the open group (25.8 vs. 26.5, *p* = 0.03). The endoscopy group had a significantly higher proportion of male patients compared to the open group (58% vs. 55%, *p* = 0.02). The Charlson Comorbidity Index was also significantly higher in the endoscopy group (4.44 ± 1.62 vs. 2.78 ± 0.92, *p* = 0.02).

Preoperatively, radicular pain (36.6%) and focal back (34.1%) pain were the most reported symptoms, followed by weakness (17.0%) or numbness and tingling (9.8%). Patients in the endoscopy group reported a significantly shorter duration of symptoms compared to the open group (6.3 months vs. 8.1 months, *p* < 0.001). Symptom prevalence also significantly varied between groups (*p* = 0.01). Radiculopathy was more common in the endoscopy group compared to the open group (78% vs. 72%), whereas back pain was more prevalent in the open group compared to the endoscopy group (20% vs. 15%). Weakness was reported at similar rates in both endoscopic and open groups (7% vs. 8%).

The targeted levels for surgery included most frequently the L4–L5 disk (50.2%), followed by L5–S1 (37.7%), L3–L4 (8.0%), and lastly L2–L3 (2%). There was a significant difference in the distribution of surgical levels between the endoscopy and open groups (*p* < 0.001). The endoscopic and open interventions were both performed mostly at L4–L5, followed by L5–S1, then L3–L4. However, there was a higher proportion of endoscopy cases performed at L4–L5 than in the open cases (65% vs. 58%), as well as at L3–L4 (8% vs. 5%). There was a lower proportion of endoscopy cases performed at L5–S1 than in the open cases (30% vs. 37%).

### 3.2. Postoperative Outcomes and Complication Rates

Post-operative VAS Leg Pain scores were significantly improved in the endoscopic compared to the open group, both at 1-day and 1-year post-operative time points (*p* < 0.001, Table 2). With respect to complications, dural tears occurred in 1.0% of ELMD cases compared to 2.2% in the open group, while wound infections occurred in 0.9% and 3.7%, respectively. Although these differences did not reach statistical significance, both trends favored the endoscopic approach. Recurrent disk herniation was significantly lower following ELMD (4.8% vs. 5.2%, *p* < 0.001), representing the most consistent outcome advantage. Reported revision rates were correspondingly lower in the ELMD group, reflecting reduced recurrence and fewer major complications.

### 3.3. MIS vs. Endoscopic Diskectomy

Comparing patients undergoing MIS non-endoscopic discectomy and patients undergoing ELMD, there was no significant difference in pre-operative patient characteristics, including gender, age, BMI, or symptoms duration—markedly differing from comparisons between ELMD and open surgery groups. ELMD was more frequently performed with lower lumbar herniated disks, including at L4-L5 and L5-S1 levels. VAS leg pain trended towards lower scores in the endoscopic group both on post-op day 1 and at 1 year follow-up, but this was not statistically significant, likely from underpowered MIS data (*p* = 0.09). Comparative data may be reviewed in Table 3.

### 3.4. FELD: Interlaminar vs. Transforaminal

Amongst patients who underwent FELD, 483 underwent interlaminar approaches to diskectomy while 1,083 underwent transforaminal; the approach was not reported for the remaining patients in our analysis. There was no significant difference in pre-operative patient characteristics between groups aside from older age in the interlaminar group (46.8 yo vs. 44.9, *p* = 0.006). There was also no significant difference in post-operative VAS leg pain (*p* = 0.07) or rates of complication infection (*p* = 0.4) or disk recurrence (*p* = 0.43), although rates of dural tear (1.4% vs. 0.6%, *p* = 1.65) trended higher in the interlaminar group (Table 4). Transforaminal approaches were more frequently utilized for L4–L5 diskectomies (57.3% vs. 42.9%, *p* < 0.001) while interlaminar approaches were more commonly used at the L5–S1 level (49.9% vs. 36.2%, *p* < 0.001). There was no significant difference in pre-operative symptomatology between groups, but patients undergoing interlaminar endoscopic diskectomy trended towards having higher rates of motor weakness and sensory loss at presentation (Table 5).

### 3.5. FELD and BELD

The large majority of ELMD patients included in this analysis underwent FELD (1,428) compared to BELD (138). There was no significant difference in pre-operative gender, age, or BMI. However, patients undergoing BELD had significantly longer symptom duration (39.3 wks vs. 84 days). There was no difference in VAS leg outcome (*p* = 0.11) or rates of complication (recurrence, *p* = 0.25; dural tear, *p* = 0.08; infection, *p* = 0.33, Table 6). The level of pathology treated was also not significantly different between groups, with similar rates of diskectomy performed from L2–L3 to L5-S1 between techniques. However, patients undergoing BELD had higher rates of pre-operative motor weakness (16.7% vs. 8.5%, *p* = 0.002) and hyporeflexia (6.5% vs. 3.1%, *p* = 0.04, Table 7).

## 4. Discussion

The findings of this review demonstrate the clear utility of endoscopic approaches to address lumbar disk herniations. Compared to open approaches, endoscopic microdiscectomy promotes faster recovery, decreases overall hospital stay, and improves quality of life outcomes [7,98]. To our knowledge, this is the largest systematic review and meta-analysis investigating pre-operative patient characteristics and post-operative outcome data of patients undergoing endoscopic microdiscectomy compared to traditional open diskectomy and non-endoscopic MIS diskectomy. This is also the first review to focus on differences in patient characteristics and surgical targets between various endoscopic approaches. The hope is that this review will prompt further discussion of the optimal patient profile for FELD and BELD, as well as how these may impact surgical approach and technique.

Regarding patient demographics, there was a significantly larger proportion of males undergoing endoscopic surgery when compared to an open approach, as well as a generally younger population undergoing endoscopic approaches. While there are a number of possible explanations for this, the authors of this manuscript believe that this may at least partially be due to the younger patients seeking out the more “cutting edge” and minimally invasive treatment options. Indeed, younger patients tend to be more tech savvy and generally have easier access to online data, which can lead them to endoscopic specialists. Further, while a majority of all patients undergoing lumbar microdiscectomy were slightly overweight, patients who had an endoscopic surgery had a significantly lower BMI than those who had an open surgery. This finding seems somewhat paradoxical, as endoscopic surgery is ideally suited to treat obese patients. While traditional tubular MIS surgery has significant limitations with obese patients, an endoscopic surgery on low- and high-BMI patients is virtually identical. This finding is therefore more likely related to the higher proportion of younger, and likely more physically fit, patients than the older group associated with more traditional approaches. Lastly, patients who underwent endoscopic lumbar microdiscectomy had, unsurprisingly, a significantly higher Charlson Comorbidity Index than those undergoing an open procedure. This may be representative of a preference for more minimally invasive techniques, which prevent complications from surgical trauma, blood loss, and prolonged recovery time that may occur in high-risk patients during an open procedure. Indeed, full endoscopic spine surgery has been shown to be safe and tolerated in patients with a high CCI [99].

In regard to the surgical level, while L4–L5 was the most commonly targeted area in both endoscopic and open approaches, the proportion of patients undergoing microdiscectomy at L3–L4 and L4–L5 was higher in the endoscopic group; whereas, L5-S1 was more frequently targeted in the open group. These findings may be due to the fact that not all endoscopic practitioners are performing both transforaminal and interlaminar endoscopic surgery. The interlaminar approach is what “unlocks” all spinal disk pathology to be treated endoscopically. This theory is further supported by the fact that the transforaminal endoscopic discectomy approach at L5-S1 is infrequently utilized, given challenges posed by a high iliac crest, sacral ala, large transverse process, wide facet joint, and narrow foramen [100]. Amongst patients undergoing endoscopic surgery, this point is represented by a higher proportion of interlaminar cases being performed at L5-S1, whereas transforaminal discectomies were more frequently performed at more cranial levels. This finding is not surprising and likely related to the larger interlaminar window at lower lumbar segments [101].

Patients undergoing endoscopic microdiscectomy experienced less leg pain in the immediate postoperative period and at 1 year compared to open surgery. Additionally, while comparisons in VAS leg pain outcomes were not statistically significant compared to patients undergoing non-endoscopic MIS surgery, there was a clear trend towards lower leg pain immediately post-operatively that persisted to one-year follow-up in the endoscopic group. This could reflect the reduced muscle and tissue disruption and minimal bony work with endoscopy, possibly leading to faster early recovery and equally effective nerve decompression long-term. Additionally, decreased tissue disruption may result in a lesser amount of scar tissue formation, which may contribute to recurrent radiculopathy in more invasive surgeries. Additionally, lower rates of recurrent disk were seen in patients undergoing endoscopic diskectomy compared to open. The etiology of this difference remains unclear, but several existing propositions regarding the advantages of the endoscopic approach may explain this. One theory is reduced tissue disruption and iatrogenic micro-instability from surgery with the endoscopic approach, which may be a biomechanical factor leading to increased rates of recurrent disks in those undergoing open surgery [102]. Similarly, less radical discectomy, smaller annular defect profile, and improved endplate preservation may also be contributing factors [103]. Of course, selection bias with patients undergoing open discectomy being of higher age and BMI may be confounding variables, and therefore, future multivariate analysis is needed to further elucidate these discrepancies [102].

Comparing BELD vs. FELD approaches, there was a significantly longer duration of symptoms in the BELD group, with patients undergoing BELD having had symptoms for 39 weeks on average compared to 84 days in the FELD group. This may suggest the presence of more long-standing pathology, such as neurogenic claudication, facet arthropathy, calcified disks, or high-grade migrations in the BELD group. In general, the pathologies associated with central stenosis may cause a practitioner to embrace BELD, which some view as more efficient when significant bony work is required. Interestingly, patients undergoing BELD also more frequently presented with motor weakness and hyporeflexia, again suggesting more longstanding pathology. However, while the complexity and chronicity of pathology may have an impact on patient selection for BELD vs. FELD, the level of pathology seemingly does not. Both BELD and FELD approaches were utilized throughout all segments of the lumbar spine without a significant difference in the level of surgery. A brief remark is pertinent regarding the theoretical differences in degree of tissue disruption between FELD and BELD, with many practitioners suggesting the latter may be more invasive given the need for two incisions, two working ports, and creation of a potential space at the intersection of the viewing and working ports [104]. While there may exist a physiologic difference in the degree of tissue disruption between the two approaches, our results seem to suggest this may not translate into a clinically meaningful difference, as patients undergoing FELD and BELD exhibited similar complication rates and outcome scores. Of course, this may be a result of an underpowered analysis, as FELD articles dramatically outnumbered BELD; indeed, further investigation is needed.

## 5. Limitations

One limitation of this study is its focus on reporting differences in patient characteristics of those undergoing ELD vs. open microdiscectomy rather than investigating the underlying factors driving the selection of endoscopic surgery for these specific patients. In that sense, this publication is reporting the current profile of patients undergoing these procedures, rather than the ideal profile of these patients. It is unclear how the variables explored in this review, such as level of pathology, gender, age, BMI, symptom duration, and comorbidity, influence a surgeon’s suggestion of an endoscopic over an open approach. Furthermore, other considerations, such as the complexity of pathology and patient preferences and risk tolerance, may also be factored into the final decision of proceeding with an endoscopic lumbar microdiscectomy. Key areas of focus for future research should include the identification of which patient characteristics most influence a surgeon’s decision, as well as factors that predict successful outcomes for patients. A phase 2 implementation of this study will utilize a modified Delphi approach to survey a diverse, global team of spine specialists and reach a consensus regarding the ideal patient profile for endoscopic spinal procedures.

Another important limitation of this analysis is that patients undergoing endoscopic lumbar discectomy were generally younger, with lower BMI and different comorbidity profiles compared to those in the open surgery cohort. These baseline demographic and clinical differences may represent selection bias and could independently influence outcomes, thereby confounding direct comparisons between groups. For example, younger patients with lower BMI may recover faster regardless of surgical approach, while higher comorbidity indices may reflect a tendency to preferentially offer less invasive procedures to higher-risk patients. Future prospective randomized trials with better-matched cohorts will be necessary to disentangle these confounding factors and determine the true comparative effectiveness of endoscopic versus open microdiscectomy.

The comparison in outcomes between endoscopic microdiscectomy and MIS non-endoscopic diskectomy is prudent. Unfortunately, the literature evaluating this remains sparse. While the review attempted to provide insight into this question, we must recognize that comparisons were likely underpowered. Additionally, this review pooled data across multiple endoscopic techniques, including FELD, BELD, transforaminal, and interlaminar approaches. Differences in surgical technique, surgeon expertise, and learning curve effects likely contributed to heterogeneity that could not be fully accounted for in the present analysis. Differences between FELD and BELD outcomes may also reflect learning curve effects rather than inherent technical limitations. Recent works suggest BELD achieves proficiency in fewer cases than FELD, and experience with one approach can shorten the learning curve of the other [105]. Still, results showing a trend toward lower VAS leg pain scores in the endoscopy group are promising. Future randomized controlled studies evaluating MIS versus endoscopic approaches to discectomy in otherwise equivocal cases, echoing prior comparisons between MIS and open approaches, are needed.

Lastly, the majority of included studies originated from Asia, with relatively few from Europe or North America. This geographic concentration may limit the global generalizability of our findings, as regional differences in patient demographics, surgical training, and healthcare systems could influence outcomes.

## 6. Conclusions

This review highlights the pre-operative characteristics of patients currently being selected for ELD: younger age, male prevalence, lower BMI, higher comorbidity index, and presence of radiculopathy. It also highlights improved recovery, longer relief of pain, and lower rates of recurrent disk herniation compared to open surgery. These results also seem to suggest improved outcomes versus non-endoscopic MIS. While this report sought to evaluate pre-operative characteristics and post-operative outcome of patients currently being selected for ELD, these results will inform a phase 2 modified Delphi study to further elucidate ideal characteristics of surgical candidates and develop a management algorithm for endoscopic lumbar surgery.

## Figures and Tables

**Table 1:** Endoscopy vs. open characteristics.

	Endoscopy Group	95% CI	Open Group	95% CI	*p*-Value
	(N = 2074)		(N = 1167)		
**Gender (Male %)**	58%	56–60%	55%	52–58%	**0.02**
**Age (Mean)**	48.2 years	47.5–48.9	52.7 years	51.3–54.1	**<0.001**
**BMI (Mean)**	25.8	25.5–26.1	26.5	26.1–26.9	**0.03**
**Duration of Symptoms**	6.3 months	6.0–6.6	8.1 months	7.7–8.5	**<0.001**
**Charlson Comorbidity Index**	4.44	±1.62 (SD)	2.78	±0.92 (SD)	**0.02**
**Level of Surgery**					
*L3–L4*	8% (166)	7–9%	5% (58)	4–6%	**<0.001**
*L4–L5*	62% (1286)	60–64%	58% (677)	55–61%	
*L5–S1*	30% (622)	28–32%	37% (432)	34–40%	
**Symptom Prevalence**					
*Radiculopathy*	78% (1618)	76–80%	72% (840)	69–75%	**0.01**
*Back Pain*	15% (311)	13–17%	20% (233)	18–22%	
*Weakness*	7% (145)	6–8%	8% (93)	6–10%	

**Table 2:** Post-operative outcomes of endoscopy vs. open microdiscectomy.

VAS Leg Pain	Endoscopy Group	Open Group	95% CI (Endoscopy vs. OD)	*p*-Value
*1 day*	1.7 ± 1.3	3.1 ± 2.2	Δ = −1.4 [−1.7 to −1.1] (1 day)	<0.001 (*t*-test)
*1 year*	1.2 ± 0.6	2.1 ± 1.5		
**Complications**				
*Dural Tear*	Dural Tear: 13 (1.0%)	Dural Tear: 9 (2.2%)	OR: 0.91 [0.55–1.51] (Recurrence)	0.71 (Fisher’s)
*Wound Infection*	Infection: 12 (0.9%)	Infection: 15 (3.7%)	OR: 0.71 [0.33–1.53] (Recurrence)	0.38 (Fisher’s)
*Recurrent disk*	Recurrence: 63 (4.8%)	Recurrence: 21 (5.2%)	OR: 0.19 [0.11–0.31] (Recurrence)	<0.001 (Fisher’s)

**Table 3:** Pre-operative and post-operative comparisons of endoscopic versus MIS cohort.

Variable	MIS Group (N = 249)	Endoscopy Group (N = 1317)	95% CI (MED vs. Other)	*p*-Value
**Gender (Male:Female)**	146 M:103 F (58.6% M)	725 M:592 F (55.1% M)		0.24
**Age (Years)**	44.9 ± 12.6	45.8 ± 11.3		0.38
**BMI**	27.1 ± 2.8 (reported in 70% of MED cases)	26.8 ± 3.1 (reported in 68% of cases)		0.52
**Duration of Symptoms**	82 ± 16.3 days	84 ± 12.9 days (PTED), 85 ± 10.6 days (PEID)		0.41
**Level of surgery**				
*L2/L3*	17 (6.8%)	28 (2.1%)	[1.72–6.11]	**<0.001**
*L3/L4*	23 (9.2%)	48 (3.6%)	[1.53–4.28]	**0.001**
*L4/L5*	113 (45.4%)	284 (21.6%)	[1.68–2.63]	**<0.001**
*L5/S1*	96 (38.6%)	231 (17.5%)	[1.73–2.82]	**<0.001**
**Symptom prevalence**				
*Radiculopathy*	249 (100%)	1317 (100%)	[1.00–1.00]	1
*Motor Weakness*	31 (12.4%)	121 (9.2%)	[0.91–2.00]	0.13
*Sensory Loss*	190 (76.3%)	893 (67.8%)	[1.02–1.24]	**0.02**
*Hyporeflexia*	13 (5.2%)	45 (3.4%)	[0.82–2.86]	0.18
**Post-operative Complications**				
*disk Recurrence*	10 (4%)	63 (4.8%)	OR: 0.83 [0.41–1.66]	0.59 (Fisher’s)
*Dural Tear*	0	13 (1%)	OR: 0.21 [0.01–3.476]	0.27
*Infection*	3 (1.2%)	12 (0.9%)	OR: 1.28 [0.36–4.58]	1.28
**VAS Leg Pain (Post-Op)**	1.9 ± 1.5 (1 day)	1.7 ± 1.3 (1 day)	Δ = 0.2 [−0.1–0.5] (1 day)	0.09
	1.4 ± 0.8 (1 year)	1.2 ± 0.6 (1 year)	Δ = 0.2 [−0.1–0.5] (1 day)	

**Table 4:** Pre-operative and post-operative comparisons of endoscopic interlaminar versus endoscopic transforaminal approaches.

Variable	Interlaminar Group (N = 483)	Transforaminal Group (N = 1083)	95% CI (Interlaminar Vs. Transforaminal)	*p*-Value
**Gender (Male:Female)**	253 M:230 F (52.4% M)	603 M:480 F (55.7% M)		0.18
**Age (Years)**	46.8 ± 11.2	44.9 ± 12.4		**0.006**
**BMI**	26.5 ± 2.9 (reported in 70% of cases)	26.8 ± 3.1 (reported in 66% of cases)		0.09
**Duration of Symptoms**	85 ± 10.6 days	84 ± 12.9 days		0.28
**VAS Leg Pain (Post-Op)**	1.8 ± 1.4 (1 day)	1.6 ± 1.2 (1 day)	Δ = 0.2 [−0.1–0.5] (1 day)	0.07
	1.3 ± 0.7 (1 year)	1.1 ± 0.6 (1 year)		
**Complications**	Recurrence: 22 (4.6%)	Recurrence: 41 (3.8%)	OR: 1.24 [0.73–2.10] (Recurrence)	0.43 (Fisher’s)
	Dural Tear: 7 (1.4%)	Dural Tear: 6 (0.6%)	OR: 2.70 [0.90–8.05] (Recurrence)	1.65
	Infection: 5 (1.0%)	Infection: 7 (0.6%)	OR: 1.65 [0.36–5.22] (Recurrence)	0.4

**Table 5:** Surgical level and pre-operative symptomatology of interlaminar versus transforaminal endoscopic diskectomy.

Level	Interlaminar Group (N = 483)	Transforaminal Group (N = 1083)	Risk Ratio (Interlaminar Vs. Transforaminal)	95% CI	*p*-Value
*L2/L3*	8 (1.7%)	20 (1.8%)	0.91	[0.40–2.08]	0.83
*L3/L4*	12 (2.5%)	36 (3.3%)	0.75	[0.39–1.45]	0.39
*L4/L5*	207 (42.9%)	621 (57.3%)	0.75	[0.64–0.88]	**<0.001**
*L5/S1*	241 (49.9%)	392 (36.2%)	1.38	[1.18–1.61]	**<0.001**
**Symptom**	**Interlaminar Group (N = 483)**	**Transforaminal Group (N = 1083)**	**Risk Ratio (Interlaminar Vs. Transforaminal)**	**95% CI**	***p*-value**
*Radiculopathy*	483 (100%)	1,083 (100%)	1	[1.00–1.00]	1
*Motor Weakness*	51 (10.6%)	93 (8.6%)	1.23	[0.88–1.72]	0.22
*Sensory Loss*	341 (70.6%)	722 (66.7%)	1.06	[0.96–1.17]	0.24
*Reflex Loss*	25 (5.2%)	37 (3.4%)	1.52	[0.92–2.51]	0.1

**Table 6:** Pre-operative and post-operative comparisons of BELD and FELD groups.

Variable	BELD (N = 138)	FELD (N = 1428)	95% CI	*p*-Value
**Gender (Male:Female)**	79 M:59 F (57.2% M)	777 M:651 F (54.4% M)		0.52
**Age (Years)**	47.1 ± 10.4	45.2 ± 12.1		0.08
**BMI**	26.9 ± 2.6 (reported in 75% of cases)	26.7 ± 3.2 (reported in 67% of cases)		0.45
**Duration of Symptoms**	39.3 ± 44.1 weeks	84 ± 12.9 days (PTED), 85 ± 10.6 days (PEID)		**<0.001**
**VAS Leg Pain (Post-Op)**	2.0 ± 1.5 (1 day)	1.7 ± 1.3 (1 day)	Δ = 0.3 [−0.1–0.7] (1 day)	0.11
	1.2 ± 0.7 (1 year)	1.1 ± 0.6 (1 year)		
**Complications**	Recurrence: 8 (5.8%)	Recurrence: 55 (3.9%)	OR: 1.56 [0.73–3.35] (Recurrence)	0.25 (Fisher’s)
	Dural Tear: 3 (2.2%)	Dural Tear: 10 (0.7%)	OR: 3.22 [0.88–11.86] (Recurrence)	0.08
	Infection: 2 (1.4%)	Infection: 10 (0.7%)	OR: 2.15 [046–9.91] (Recurrence)	0.33

**Table 7:** Operative level and pre-operative symptomology in FELD and BELD.

Level	BELD (N = 138)	FELD (N = 1428)	Risk Ratio (Biportal vs. Other)	95% CI	*p*-Value
*L2/L3*	3 (2.2%)	25 (1.8%)	1.24	[0.37–4.15]	0.73
*L3/L4*	7 (5.1%)	41 (2.9%)	1.76	[0.79–3.93]	0.16
*L4/L5*	70 (50.7%)	784 (54.9%)	0.92	[0.72–1.18]	0.51
*L5/S1*	53 (38.4%)	583 (40.8%)	0.94	[0.71–1.24]	0.67
**Symptom**					
*Radiculopathy*	138 (100%)	1428 (100%)	1	[1.00–1.00]	1
*Motor Weakness*	23 (16.7%)	121 (8.5%)	1.97	[1.28–3.03]	**0.002**
*Sensory Loss*	94 (68.1%)	969 (67.9%)	1.01	[0.86–1.18]	0.93
*Hyporeflexia*	9 (6.5%)	44 (3.1%)	2.11	[1.03–4.31]	**0.04**

## Data Availability

The data supporting the findings of this study are available within the article and upon request. All data were extracted from previously published studies identified through PubMed, Embase, Scopus, and hand searches, as detailed in the Section 2. No new patient-level data were generated. The list of included studies and extracted datasets used in the meta-analysis is provided.

## Abbreviations

The following abbreviations are used in this manuscript:

PRISMA	Preferred Reporting Items for Systematic Reviews and Meta-Analyses
FELD	Full Endoscopic Lumbar Discectomy
BELD	Biportal Endoscopic Lumbar Discectomy
ELMD	Endoscopic Lumbar Microdiscectomy
MIS	Minimally Invasive Spine Surgery
VAS	Visual Analog Scale
BMI	Body Mass Index
